# Reply to the letter by Kondo et al: Mechanisms for new‐onset atrial fibrillation in patients with acute coronary syndrome

**DOI:** 10.1002/joa3.12470

**Published:** 2020-12-10

**Authors:** Mizuyoshi Nagai, Tomonori Itoh, Yoshihiro Morino

**Affiliations:** ^1^ Division of Cardiology Department of Internal Medicine Iwate Medical University Yahaba Japan

To the Editor,

We read with great interest the "Letter to the Editor" by Kondo et al titled “New onset atrial fibrillation after atrial ischemia.”^1^

We appreciate your valuable feedback. We have the following comments about your response.

We hold the same opinion that atrial ischemia may play a key role in the development of new‐onset atrial fibrillation (NOAF) after acute coronary syndrome (ACS). However, we did not consider atrial ischemia in our study.^2^ As you have pointed out, no new reports appear to exist regarding NOAF following ACS. Recently, Khalfallah et al reported that a right coronary artery (RCA) culprit vessel was one of the independent predictors of NOAF.^3^ They also reported that ST‐elevated myocardial infarction, advanced age, hypertension, LAVI >34 mL/m^2^, E/e′ ratio >12, and heart failure were independent prognostic factors.

Thus, NOAF may be caused by multiple factors. These factors, as mentioned by Kondo et al, may be as follows: (a) structural and/or electrophysiological abnormalities change atrial tissue to facilitate abnormal impulse formation and/or propagation; and (b) a combination of multiple factors, such as genetic components, heart failure, atrial stretch, and ischemia, sympathovagal influences, inflammation, and fibrosis.

Because atrial fibrillation may be caused by atrial ischemia, as stated by Kondo et al, ischemia of RCA, main source of atrial branch, may result in atrial fibrillation. In contrast, an atrial pressure rise associated with left ventricular end‐diastolic pressure caused by heart failure is also considered a common cause.

Hence, NOAF may be also caused by anterior myocardial infarction in addition to inferior myocardial infarction. Recently, we discovered a new factor associated with NOAF. Although it has not been published in the literature yet, our institution reported that the coronary slow flow after PCI was an independent predictor of NOAF in patients with ACS. (HR = 3.17, 95% CI: 1.47‐6.82, *P* = .003: Niiyama et al, Abstract: P5574, *European Heart Journal*. 2018:39(Supplement), 1163).

Coronary slow flow is a factor in left ventricular remodeling, especially in anterior myocardial infarction, and may cause elevated left ventricular end‐diastolic pressure. On the other hand, if the proximal right coronary artery is the culprit lesion, NOAF may easily occur owing to the proximal occlusion complicated with right ventricular infarction because the right atrial pressure rises following right ventricular end‐diastolic pressure rise. It may worsen by coronary slow flow that occurs after PCI, especially in the proximal right coronary artery which is large in diameter and rich in thrombus.

Thus, there are multiple factors affecting the onset of NOAF, including atrial ischemia in inferior myocardial infarction, increased left ventricular end‐diastolic pressure in anterior myocardial infarction, and coronary slow flow in both cases. Further evidence is required to clarify the pathogenic relationship between atrial fibrillation and the mechanism of its onset in patients with ACS.

## CONFLICT OF INTEREST

The authors have no conflicts of interest directly relevant to the content of this article.
